# Acknowledgement of manuscript reviewers 2014

**DOI:** 10.1186/s12970-015-0071-1

**Published:** 2015-04-02

**Authors:** Jose Antonio, Douglas Kalman, Richard Kreider

**Affiliations:** ᅟ, Davie, USA; ᅟ, Miami, USA; ᅟ, College Station, USA

## Abstract

The editors of *Journal of the International Society of Sports Nutrition* would like to thank all our reviewers who have contributed to the journal from Volume 11 (2014).

**Michelle Adams**

United States of America

**Jose Antonio**

United States of America

**Alan Aragon**

United States of America

**Luis Aragon**

Costa Rica

**Shawn Arent**

United States of America

**Sainath Babu**

United States of America

**Audrey Baguet**

Belgium

**Derek Ball**

United Kingdom

**Laurent Bannock**

United Kingdom

**Matthew Barnes**

New Zealand

**Katherine Beals**

United States of America

**Dan Benardot**

United States of America

**Sandeep Bhale**

United States of America

**Scarlett Blandon**

United States of America

**Amanda Bonikowske**

United States of America

**Aurelien Bringard**

Switzerland

**Will Brink**

United States of America

**Erik Bustillo**

United States of America

**Bill Campbell**

United States of America

**Sara Campbell**

United States of America

**Erico Caperuto**

Brazil

**Michael Carlston**

United States of America

**Andres Carrillo**

United States of America

**Shuping Chen**

Taiwan

**Amanda Claassen-Smithers**

South Africa

**Matthew Cocks**

United Kingdom

**Adriana Coletta**

United States of America

**Scott Conger**

United States of America

**Joel Cramer**

United States of America

**Ben Dascombe**

Australia

**Inna Dumova**

United States of America

**Conrad Earnest**

United States of America

**Joan Eckerson**

United States of America

**Anya Ellerbroek**

United States of America

**Mark Faries**

United States of America

**Anthony Fisher**

United Kingdom

**Andreas Flouris**

Greece

**Mark Fogarty**

United Kingdom

**Elfego Galvan**

United States of America

**Jeffrey Godin**

United States of America

**Joanna Gromadzka-Ostrowska**

Poland

**Bruno Gualano**

Brazil

**Daniel Gwartney**

United States of America

**Michael Hartman**

United States of America

**Phillip Harvey**

United States of America

**Travis Harvey**

United States of America

**Judith Haudum**

Austria

**Jaramillo Londoño Hilda Norha**

Colombia

**Glyn Howatson**

United Kingdom

**Geoffrey Hudson**

United States of America

**Patrick Jacobs**

United States of America

**Ralf Jäger**

United States of America

**Andrew Jagim**

United States of America

**Xanne Janse De Jonge**

Australia

**Douglas Kalman**

United States of America

**Bettina Karsten**

United Kingdom

**Kristina Kendall**

United States of America

**Wesley Kephart**

United States of America

**Chad Kerksick**

United States of America

**Arie Kies**

Netherlands

**Andrea Kirk**

United States of America

**Susan Kleiner**

United States of America

**Beat Knechtle**

Switzerland

**Richard Kreider**

United States of America

**Jamie Krzykowski**

United States of America

**Paul La Bounty**

United States of America

**Anna Lepeley**

United States of America

**Kyle Levers**

United States of America

**Vivienne Lewis**

United States of America

**Chris Lockwood**

United States of America

**Hector Lopez**

United States of America

**Lonnie Lowery**

United States of America

**Ryan Lowery**

United States of America

**James Lugo**

United States of America

**Shannan Lynch**

United States of America

**Andrew Mckune**

South Africa

**Ron Mendel**

United States of America

**Antti A Mero**

Finland

**Christopher Mobley**

United States of America

**Jordan Moon**

United States of America

**Taro Murakami**

Japan

**Michael Nelson**

United States of America

**Georg Neumann**

Germany

**Jonathan Oliver**

United States of America

**Cheong Hwa Ooi**

Malaysia

**Michael Ormsbee**

United States of America

**Hidenori Otani**

Japan

**Corey Peacock**

United States of America

**Sunita Potgieter**

South Africa

**Jonato Prestes**

Brazil

**Martin Purpura**

United States of America

**Christoph Raschka**

Germany

**Nicholas Ratamess**

United States of America

**John Rathmacher**

United States of America

**Michael Roberts**

United States of America

**Vanessa Rodrigues Vilela**

Brazil

**F.Nese Sahin**

Turkey

**Steve Schmitz**

United States of America

**Brad Schoenfeld**

United States of America

**Neil Schwarz**

United States of America

**John Seifert**

United States of America

**Piero Sestili**

Italy

**Andrew Shao**

United States of America

**Tobin Silver**

United States of America

**Abbie Smith-Ryan**

United States of America

**Jeffrey Stout**

United States of America

**Mark J. Tallon**

United Kingdom

**Minghua Tang**

United States of America

**Lemuel W Taylor**

United States of America

**Lisa Te Morenga**

New Zealand

**Nicholas Tiller**

United Kingdom

**Julio Tirapegui**

Brazil

**Gianluca Tognon**

Sweden

**Marie-Berengere Troadec**

France

**Benjamin Wax**

United States of America

**Shawn Wells**

United States of America

**Darryn Willoughby**

United States of America

**Jacob Wilson**

United States of America

**Abdelmoneim Younis**

United States of America

**Yang Zhang**

China

**Hassane Zouhal**

France

